# Association of glomerular filtration rate slope with timely creation of vascular access in incident hemodialysis

**DOI:** 10.1038/s41598-021-92359-w

**Published:** 2021-06-23

**Authors:** Lee-Moay Lim, Ming-Yen Lin, Shang-Jyh Hwang, Hung-Chun Chen, Yi-Wen Chiu

**Affiliations:** 1 Division of Nephrology, Department of Internal Medicine, Kaohsiung Medical University Hospital, Kaohsiung Medical University, 100 Tzyou First Road, Sanmin District, Kaohsiung, 80708 Taiwan; 2 Faculty of Medicine, College of Medicine, Kaohsiung Medical University, Kaohsiung, Taiwan; 3 Faculty of Renal Care, College of Medicine, Kaohsiung Medical University, Kaohsiung, Taiwan

**Keywords:** End-stage renal disease, Haemodialysis, Outcomes research, Health care

## Abstract

The factors associated with the timely creation of distal vascular access for hemodialysis initiation are unclear. We aimed to explore the association between the slope of estimated glomerular filtration rate (eGFR) and the successful usage of vascular access upon hemodialysis initiation. This single center retrospective cohort study enrolled chronic kidney disease patients who undertook a multidisciplinary care program from 2003 to 2016. Using eGFR slope as predictor, we evaluated the vascular access created timely upon hemodialysis initiation. Among the 987 patients, vascular access was created at a median eGFR of 5.8 min/ml/1.73 m^2^, with a median duration of 3.1 months before hemodialysis. The proportions of vascular access created timely, created not timely (vascular access immature), and not created were 68.5%, 8.8%, and 22.7%, respectively. There was a significant negative association of eGFR upon vascular access creation with eGFR slope (r = − 0.182, P < 0.001). The fastest eGFR slope patients (the first quartile or < − 10 min/ml/1.73 m^2^/year) had the lowest percentage of vascular access created timely. In the multivariable logistic regression analysis, only higher eGFR upon vascular access creation (P = 0.001) and eGFR slope (P = 0.009) were significantly associated with vascular access created timely. The adjusted odds ratios of each quartile of eGFR slopes for vascular access created timely were 0.46 (95% confidence interval 0.27–0.86), 1.30 (0.62, 2.72), 1.00 (reference), and 0.95 (0.48–1.87), respectively. eGFR slope is associated with the timely creation of vascular access for the initiation of hemodialysis in a reverse-J-shaped pattern and may help determine the time of vascular access creation.

## Introduction

The timely creation of distal vascular access (VA) upon initiation of dialysis has been a major recommendation in existing guidelines for the planned initiation of hemodialysis (HD)^1,2^, based on the evidence that VA has lower complications and cost, and improved survival^3^. Unplanned initiation of HD leads to additional burdens on healthcare systems as well as adverse outcomes^4^. However, an updated report from the Dialysis Outcomes and Practice Patterns study and many others still showed that numerous countries had more than 50% of patients using a temporary catheter upon initiation of HD^5–7^. Many factors have been proposed to explain the gap mentioned above, including mainly late referral, no disease insight, not being under regular pre-dialysis care, and an unpredicted clinical course^8–10^. To overwhelm this gap, the National Kidney Foundation-Kidney Disease Outcomes Quality Initiative (NKF-KDOQI) guideline suggests starting patient education on all modalities of kidney replacement options at an estimated glomerular filtration rate (eGFR) of 30 mL/min/1.73 m^2^. It further recommends referral for VA assessment and subsequent creation when the eGFR is 15–20 mL/min/1.73 m^2^, given that it is unlikely to predict the time of dialysis initiation^11^. In past decades, multidisciplinary chronic kidney disease (CKD) care programs have emerged and resulted in more effective prescriptions, a lower renal progression rate, a decrease in temporary dialysis catheter usage, and lower medical expenses^12–16^. Under the care of such a program, later referral and regular monitoring of renal progression were more under control; this made it easier to evaluate the time of determining when to create VA and how it affected the initiation of HD without using a central venous catheter. Thus, we conducted this retrospective study to explore the factors associated with the timely preparation of VA creation in a CKD population undertaking a multidisciplinary CKD care program, focusing on the renal progression rate by testing the hypothesis that slower eGFR slope is associated with better VA creation timely.

## Methods

### Study design

This retrospective cohort study included CKD patients who had followed up at a tertiary medical center in Taiwan from 2003 to 2016. The eligible subjects were those who had received multidisciplinary CKD care for more than 6 months and transitioned to HD. We excluded patients who had previously undergone kidney transplantation or peritoneal dialysis. The study protocol was approved by the Institutional Review Board of Kaohsiung Medical University Hospital (KMUHIRB-G(II)-20160024). Informed consent was obtained in written form and all clinical investigations were conducted according to the principles expressed in the Declaration of Helsinki.

### Multidisciplinary care program

We started a multidisciplinary CKD care program in 2003. Management and education were dependent on the stages of CKD, according to the 2002 NKF-KDOQI guidelines^17^, and the reimbursement policies of National Health Insurance (NHI). Detailed contents of this program have been described somewhere in our previous studies^12,18^. In 2006, Taiwan NHI launched a pay-for-performance program with indicators including CKD management, patient education, continuous care, remission of proteinuria, and maintenance of renal function for pre-End Stage Kidney Disease (ESKD) care^19,20^.

### Outcomes of VA preparation for HD

The introduction of renal replacement therapy (RRT) modality will be implemented for all patients in the multidisciplinary CKD care program when their eGFRs drop below 10 ml/min/1.73 m^2^. Usually, VA creation will be conducted for no longer than 2 weeks after surgeon consultation in this hospital. A cardiovascular surgeon consultation visit will be arranged before the creation with ultrasound mapping done at the same time if indicated. Two experienced cardiovascular surgeons performed all VA creation for most of our patients, with a surgical success rate of more than 95%. For fistula creation, local anesthesia was selected while general anesthesia was chosen for graft creation. In general, the surgeons performed end to side arterial anastomosis in both fistula and graft creation, and end to end venous anastomosis in graft creation. Occasionally, superficialization and transposition of deep fistula were needed^21^. In our hospital, it usually took 2 weeks from the cardiovascular surgeon referral to the VA creation.

Based on the status of VA used at the beginning of HD, we divided the study patients into three groups: VA created timely, VA created not timely, and VA not created. The group of VA created timely comprised patients using distal VA, including both fistula and graft, in the first three sessions of HD. The group of VA created not timely comprised patients using a temporary catheter for any one of the first three sessions of HD with distal VA created but immature. Meanwhile, the group of VA not created comprised patients using a temporary catheter for the first HD without distal VA created.

### eGFR calculation and slope

The eGFR in the study was calculated using the Modification of Diet in Renal Disease (MDRD) study equation^22^ and the Cockcroft and Gault (CG) formula^23^. In this hospital, physicians have access to all eGFR data when patients are attending clinical visits, and the eGFR slope is available at the same time. We defined the eGFR slope used in this study as using all eGFRs between the timing of CKD program enrollment and VA creation or starting HD—whichever came first. If there were fewer than three eGFR measurements within the aforementioned duration, we would include all eGFR measurements within 1 year prior to VA creation or starting HD—whichever came first. Through either quartile or clinical recommendations using the three cut values of − 10, − 5 and − 3 ml/min/1.73 m^2^ per year, we divided eGFR slopes into four groups for further analysis. In order to evaluate the effect of eGFR slope on VA maturation, we only included those stayed at program longer than 6 months.

### Covariates

Our register record or the medical informatics system within the hospital was used to obtain demographic information, namely age, sex, marital status (unmarried, married, or other), education level (years of education: 0–6, 7–12, equal to or more than 13), employment status (no, yes or retirement), smoking status (yes or no), underlying disease (chronic glomerulonephritis, diabetes mellitus, hypertension, or other), renal function upon timing of VA creation, types of VA for long-term dialysis (arteriovenous fistula (AVF), arteriovenous graft (AVG), or permanent catheter), renal function upon multidisciplinary CKD care program enrollment, renal function upon VA creation, renal function upon first dialysis, duration in multidisciplinary CKD care program, and time between first dialysis and shunt creation.

### Statistical analysis

The baseline characteristics were summarized as the mean and standard deviation or the median (interquartile range) for continuous variables and count (percentages) for categorical variables, respectively. Differences among groups were compared using the chi-squared test for categorical variables and one-way ANOVA, the Mann–Whitney U test or the Kruskal–Wallis test for continuous variables. To determine the significant differences in continuous variables between the groups, Fisher’s least significant difference test was applied after significant results were obtained from one-way ANOVA. The trend of VA preparation was described by the proportion of patients with their VA status upon the onset of HD, and shifts in trends were assessed using the Cochran–Armitage trend test. We used scatter plots with the Pearson correlation coefficient and test to investigate the linear associations between creatinine levels upon VA creation and eGFR slope, and eGFR by MDRD and CG equations upon VA creation and eGFR slope. Multivariable logistic regression analysis with backward selection was performed to determine factors associated with VA created timely. Statistical analyses were conducted using SAS 9.4 software (SAS Institute Inc., Cary, NC, USA). A two-sided *P* < 0.05 indicates statistical significance.

## Results

### Patient selection

**Figure 1:**
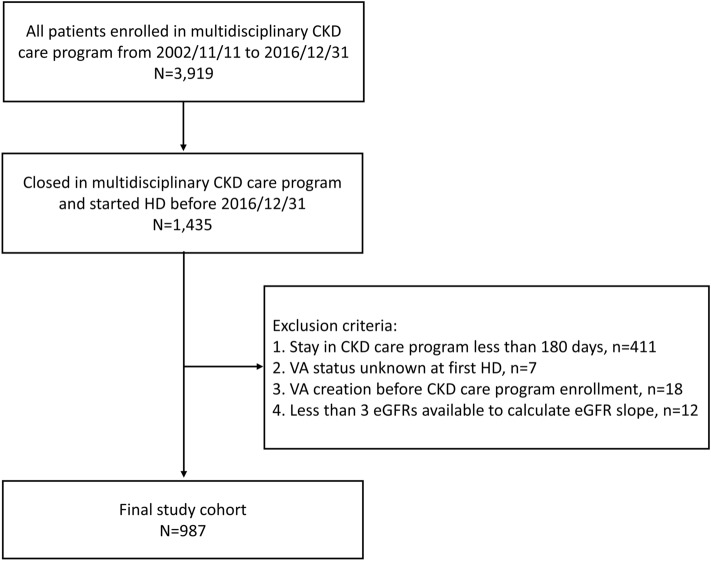
Study cohort flow chart.

Our study population was derived from a cohort of 3913 patients enrolled in the multidisciplinary CKD care program between 1 January 2003 and 31 December 2016. Of this cohort, 1435 patients entered maintenance HD. We excluded those who enrolled less than 180 days before the first HD (n = 411), whose VA status was unknown upon the first HD (n = 7), who had VA creation before CKD care program enrollment (n = 18), and those with fewer than three eGFR measurements available (n = 12). The final cohort consisted of 987 patients. Besides, 382 subjects died at the program, with 29 had VA created. Figure 1 presents a flow chart of the study cohort.

### Patient characteristics by the outcome of VA preparation

**Figure 2:**
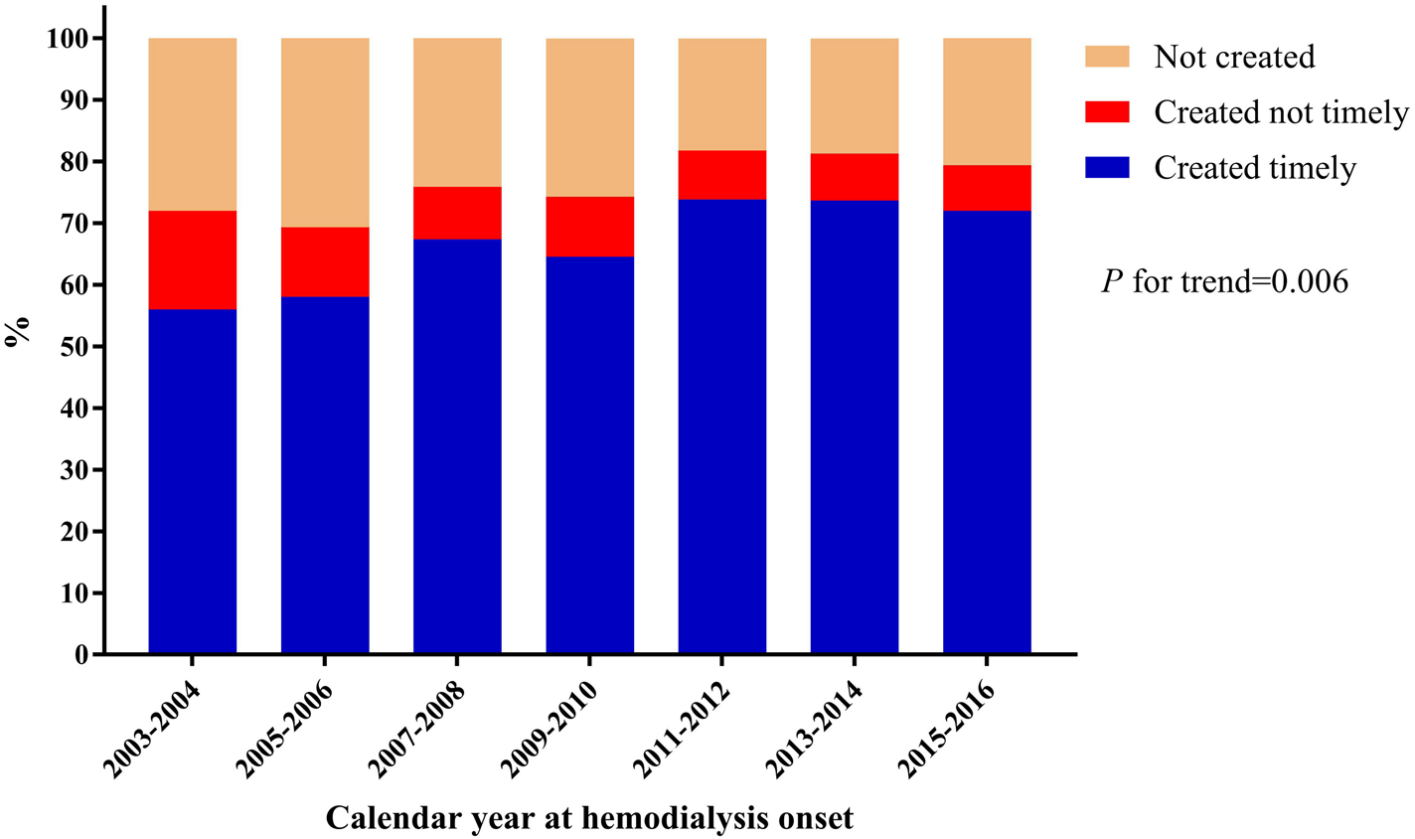
Proportion of consequences of vascular access (VA) preparation at hemodialysis onset year. A significant trend of the proportion of patients with their VA status upon the onset years of hemodialysis (*P* = 0.006) was tested by using the Cochran–Armitage trend test.

The baseline characteristics of the study cohort by consequences of VA preparation are presented in Table 1. Among these patients, 676 (68.5%) belonged to VA created timely, 87 (8.8%) belonged to VA created not timely, and 224 (22.7%) of them were in VA not created. There was no difference in demographic characteristics among the aforementioned three groups. Compared with the other groups, the VA created timely had slower CKD progression, with an eGFR slope median value of − 4.30 ml/min/year. The VA created timely had more AVF creation and, as expected, a higher eGFR level when receiving VA creation surgery. Similarly, the time between VA creation and the first HD was greater in this group, with a median of 105 days and a mean of 180 days. All groups had similar residual renal function when starting the first HD, with a median eGFR value of around 4.5 ml/min/1.73 m^2^, and the VA created not timely even had values below 4.0. Meanwhile, the VA created timely had the longest stay in the multidisciplinary CKD care program, with a median duration of more than 2 years. Figure 2 shows the proportion of patients with their VA status according to the calendar years. The comparisons among these three different time cohorts are displayed in Table 2.

**Table 1:** Clinical characteristics of incident hemodialysis patients by consequences of vascular access preparation.

	VA created timely (n = 676)	VA created not timely (n = 87)	VA not created (n = 224)
**Male sex n (%)**	367 (54.3)	38 (43.7)	113 (50.4)
**Age at CKD program enrollment (year)**	62.6 ± 11.7	63.7 ± 12.4	64.1 ± 13.1
**Age at VA creation (year)**	65.5 ± 11.8	66.0 ± 12.0	66.3 ± 13.4
**Age at first HD (year)**	65.0 ± 11.7	65.8 ± 11.9	66.6 ± 13.4
**Marital status n (%)**			
Unmarried	40 (5.9)	5 (5.7)	15 (6.7)
Married	536 (79.3)	62 (71.3)	167 (74.6)
Other	100 (14.8)	20 (23)	42 (18.8)
**Year of education n (%)**			
≤ 6	343 (50.7)	43 (49.4)	117 (52.2)
7–12	241 (35.7)	35 (40.2)	74 (33)
≥ 13	92 (13.6)	9 (10.3)	33 (14.7)
**Occupation n (%)**			
No	336 (49.7)	39 (44.8)	120 (53.6)
Yes	235 (34.8)	31 (35.6)	76 (33.9)
Retirement	105 (15.5)	17 (19.5)	28 (12.5)
**Smoke n (%)**			
No	546 (80.8)	75 (86.2)	186 (83)
Yes	130 (19.2)	12 (13.8)	38 (17)
**Primary disease n (%)**			
CGN	184 (27.2)	21 (24.1)	44 (19.6)
DM	328 (48.5)	46 (52.9)	124 (55.4)
HTN	69 (10.2)	6 (6.9)	23 (10.3)
Other	95 (14.1)	14 (16.1)	33 (14.7)
**CKD progression**			
eGFR slope (ml/min/1.73 m^2^ per year)^a^	− 4.3 (− 7.4, − 2.4)	− 5.4 (− 10.0, − 2.6)	− 5.7 (− 11.0, − 3.3)
**VA type (missing n = 13)**^**#**^** n (%)**^**a**^			
AVF	576 (85.2)	74 (85.1)	159 (75.4)
AVG	100 (14.8)	13 (14.9)	42 (19.9)
Permanent catheter	0	0	10 (4.7)
**VA used at first dialysis n (%)**^**a,b**^			
Shunt	676 (100)	0	0
Catheter	0	87 (100)	220 (98.2)
Permanent catheter	0	0	4 (1.8)
**VA created > 1 time before first HD n (%)**	9 (1.3)	1 (1.1)	0
**Renal function at multidisciplinary CKD care program enrollment**			
Cr (mg/dL)	3.8 (2.8, 5.5)	3.8 (2.7, 6.2)	3.5 (2.4, 4.9)
eGFR (MDRD) (ml/min/1.73 m^2^)	14.3 (9.3, 22.6)	14.3 (9.2, 24)	16.0 (10.9, 24.9)
eGFR (CG) (ml/min/1.73 m^2^)	15.5 (10.6, 24.2)	15.3 (9.4, 24.6)	17.6 (11.1, 27.5)
**Renal function at VA creation**			
Cr (mg/dL)^b^	8.5 (7.2, 10.3)	9.4 (7.4, 11.8)	–
eGFR (MDRD) (ml/min/1.73 m^2^)^b^	5.9 (4.8, 7.2)	5.0 (3.9, 6.7)	–
eGFR (CG) (ml/min/1.73 m^2^)^b^	6.8 (5.5, 8.4)	6.1 (4.5, 7.4)	–
**Renal function at first HD**			
Cr (mg/dL)^a^	11.2 (8.9, 13.7)	11.8 (9.3, 14.6)	10.6 (8, 13.3)
eGFR (MDRD) (ml/min/1.73 m^2^)^a,b^	4.3 (3.4, 5.5)	3.9 (3.3, 5.0)	4.6 (3.5, 5.9)
eGFR (CG) (ml/min/1.73 m^2^)^a,b^	5.2 (4.1, 6.8)	4.8 (3.9, 6.4)	5.6 (4.3, 7.4)
**Multidisciplinary CKD care program duration (days)**^a,b^	737 (417, 1440)	601 (325, 959)	509 (311, 1093)
**Time between VA creation and first HD (days)**^b^	105 (51, 226)	14 (6, 27)	–

MDRD eGFR equation: 186 $\times$ serum creatinine^−1.154^ $\times$ age^−0.203^ $\times$ 0.742 (if female)^17,22^. Cockcroft–Gault eGFR equation: [(140 − age)] $\times$ weight $\times$ 0.85 (if female)/(72 $\times$ serum creatinine), adjusted for BSA/1.73 m^2^. $BSA = weight~_{{{\text{kg}}}}^{{0.425}} \times height~_{{{\text{cm}}}}^{{0.725}} \times 0.007184$^22,23^.

Data were presented as mean (± SD) or median (interquartile range) for continuous variables and count (proportion) for categorical variables. Significant differences for continuous variables between three and two groups were tested using one-way ANOVA or the Kruskal–Wallis test and the independent t-test or the Mann–Whitney U test, respectively. However, significant differences for categorical variables between three and two groups were tested using the χ^2^ test. A two-tailed value of *P* < 0.05 was considered to be statistically significant.

*VA* vascular access, *HD* hemodialysis, *Cr* creatinine, *CKD* chronic kidney disease, *CGN* chronic glomerulonephritis, *DM* diabetes mellitus, *HTN* hypertension, *AVF* arteriovenous fistula, *AVG* arteriovenous graft, *eGFR* estimated glomerular filtration rate, *MDRD* modification of diet in renal disease, *CG* Cockcroft–Gault, *SD* standard deviation.

^#^Several cases lost their VA type because our record showed that they only used a temporary catheter at their first hemodialysis.

^a^*P* < 0.05 for the three groups.

^b^*P* < 0.05 for created timely vs. created not timely.

**Table 2:** Clinical characteristics of incident hemodialysis patients by different time cohorts.

	2003–2006 (n = 149)	2007–2010 (n = 316)	2011–2016 (n = 522)	*P* value
**Male sex n (%)**	77 (51.7)	152 (48.1)	289 (55.4)	0.1
**Age at CKD program enrollment (year)**	62.2 ± 11.1	63.8 ± 12.1	62.8 ± 12.3	0.3
**Age at VA creation (year)**	63.5 ± 11.2	66 ± 12.2	66.2 ± 12.4	0.06
**Age at first HD (year)**	63.2 ± 11.2	65.6 ± 12.3	65.9 ± 12.2	0.06
**Marital status n (%)**				0.1
Unmarried	12 (8.1)	11 (3.5)	37 (7.1)	
Married	116 (77.9)	255 (80.7)	394 (75.5)	
Other	21 (14.1)	50 (15.8)	91 (17.4)	
**Year of education n (%)**				0.005
≤ 6	85 (57.0)	175 (55.4)	243 (46.6)	
7–12	39 (26.2)	111 (35.1)	200 (38.3)	
≥ 13	25 (16.8)	30 (9.5)	79 (15.1)	
**Occupation n (%)**				< 0.001
No	68 (45.6)	195 (61.7)	232 (44.4)	
Yes	60 (40.3)	87 (27.5)	195 (37.4)	
Retirement	21 (14.1)	34 (10.8)	95 (18.2)	
**Smoke n (%)**				0.003
No	128 (85.9)	239 (75.6)	440 (84.3)	
Yes	21 (14.1)	77 (24.4)	82 (15.7)	
**Primary disease n (%)**				0.002
CGN	58 (38.9)	75 (23.7)	116 (22.2)	
DM	69 (46.3)	162 (51.3)	267 (51.1)	
HTN	7 (4.7)	34 (10.8)	57 (10.9)	
Other	15 (10.1)	45 (14.2)	82 (15.7)	
**CKD progression**				
eGFR slope (ml/min/1.73 m^2^ per year)	− 4.9 (− 9.0, − 2.2)	− 4.3 (− 7.7, − 2.3)	− 4.6 (− 8.7, − 2.7)	0.1
**VA type (missing n = 13)**^**#**^** n (%)**				0.3
AVF	125 (85.0)	262 (84.0)	422 (81.9)	
AVG	22 (15.0)	49 (15.7)	84 (16.3)	
Permanent catheter	0	1 (0.3)	9 (1.7)	
**VA used at first HD n (%)**				0.001
AV shunt	86 (57.7)	208 (65.8)	382 (73.2)	
Catheter	63 (42.3)	108 (34.2)	136 (26.1)	
Permanent catheter	0	0	4 (0.8)	
**VA created > 1 time before first HD n (%)**	1 (0.7)	4 (1.3)	5 (1)	0.9
**Renal function at CKD program enrollment**				
Cr (mg/dL)	5.3 (3.8, 7.4)	4.1 (3, 5.6)	3.3 (2.2, 4.5)	< 0.001
eGFR (MDRD) (ml/min/1.73 m^2^)	9.6 (6.7, 14.3)	13.3 (9.2, 20)	17.5 (12.1, 27.2)	< 0.001
eGFR (CG) (ml/min/1.73 m^2^)	10.9 (7.6, 17.3)	14.4 (10, 21.3)	18.9 (13.0, 29.3)	< 0.001
**Renal function at VA creation**				
Cr (mg/dL)	9.1 (6.9, 11.4)	8.6 (7.2, 10.8)	8.5 (7.3, 10.1)	0.4
eGFR (MDRD) (ml/min/1.73 m^2^)	5.6 (4.3, 7.8)	5.7 (4.5, 7.1)	5.9 (4.9, 7.2)	0.2
eGFR (CG) (ml/min/1.73 m^2^)	6.6 (4.8, 8.9)	6.2 (5, 7.9)	6.9 (5.6, 8.4)	0.002
**Renal function at first HD**				
Cr (mg/dL)	11.3 (8.7, 13.8)	10.7 (8.4, 13.8)	11.1 (9.1, 13.4)	0.8
eGFR (MDRD) (ml/min/1.73 m^2^)	4.3 (3.4, 5.6)	4.3 (3.3, 5.5)	4.4 (3.4, 5.5)	0.9
eGFR (CG) (ml/min/1.73 m^2^)	5.4 (4.1, 6.8)	5.1 (3.9, 6.7)	5.3 (4.2, 6.8)	0.2
**CKD program duration (days)**	396 (270, 625)	644 (386, 1069)	893 (461, 1758)	< 0.001
**Time between VA creation and first HD (days)**	95 (41, 211)	91 (41, 206)	95 (41, 218)	0.9

MDRD eGFR equation: 186 × serum creatinine^−1.154^ × age^−0.203^ × 0.742 (if female)^21,22^. Cockcroft–Gault eGFR equation: [(140 − age)] × weight × 0.85 (if female)/(72 × serum creatinine), adjusted for BSA/1.73m^2^. $BSA = weight~_{{{\text{kg}}}}^{{0.425}} \times height~_{{{\text{cm}}}}^{{0.725}} \times 0.007184$^22,23^.

Data were presented as mean (± SD) or median (interquartile range) for continuous variables and count (proportion) for categorical variables. Significant differences for continuous variables between groups were tested using one-way ANOVA and the Kruskal–Wallis test. However, significant differences for categorical variables between groups were tested using the χ^2^ test. A two-tailed value of *P* < 0.05 was considered to be statistically significant.

*VA* vascular access, *HD* hemodialysis, *CKD* chronic kidney disease, *CGN* chronic glomerulonephritis, *DM* diabetes mellitus, *HTN* hypertension, *AVF* arteriovenous fistula, *AVG* arteriovenous graft, *eGFR* estimated glomerular filtration rate, *Cr* creatinine, *MDRD* modification of diet in renal disease, *CG* Cockcroft–Gault.

^#^Several cases lost their VA type because we our record showed that they only used a temporary catheter at their first hemodialysis.

### eGFR slope and timely VA creation

**Figure 3:**
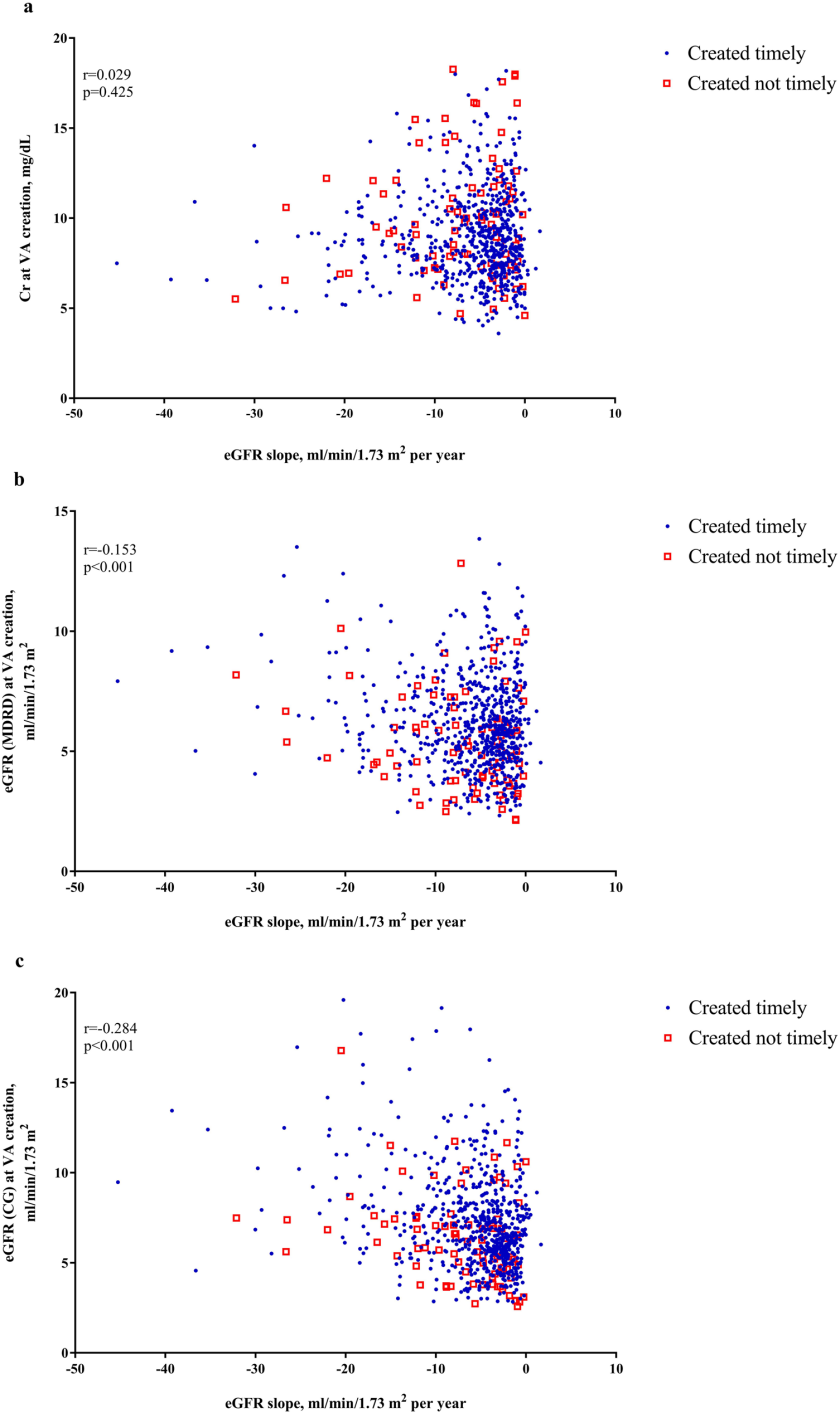
Correlation between renal function at vascular access (VA) creation and estimated glomerular filtration rate (eGFR) slope by VA created timely or not timely (n = 763). Renal function expressed by creatinine (Cr) (**a**), estimated glomerular filtration rate (eGFR) by modification of diet in renal disease (MDRD) (**b**) calculated by the equation: 186 $\times$ serum creatinine^−1.154^ $\times$ age^−0.203^ $\times$ 1.212 (if patient is black) $\times$ 0.742 (if female)^22^, and eGFR by Cockcroft–Gault (CG) (**c**) calculated by the equation: [(140 − age)] $\times$ weight $\times$ 0.85 (if female)/(72 $\times$ serum creatinine), adjusted for BSA/1.73 m^2^. $BSA = weight~_{{{\text{kg}}}}^{{0.425}} \times height~_{{{\text{cm}}}}^{{0.725}} \times 0.007184$^23^.

**Figure 4:**
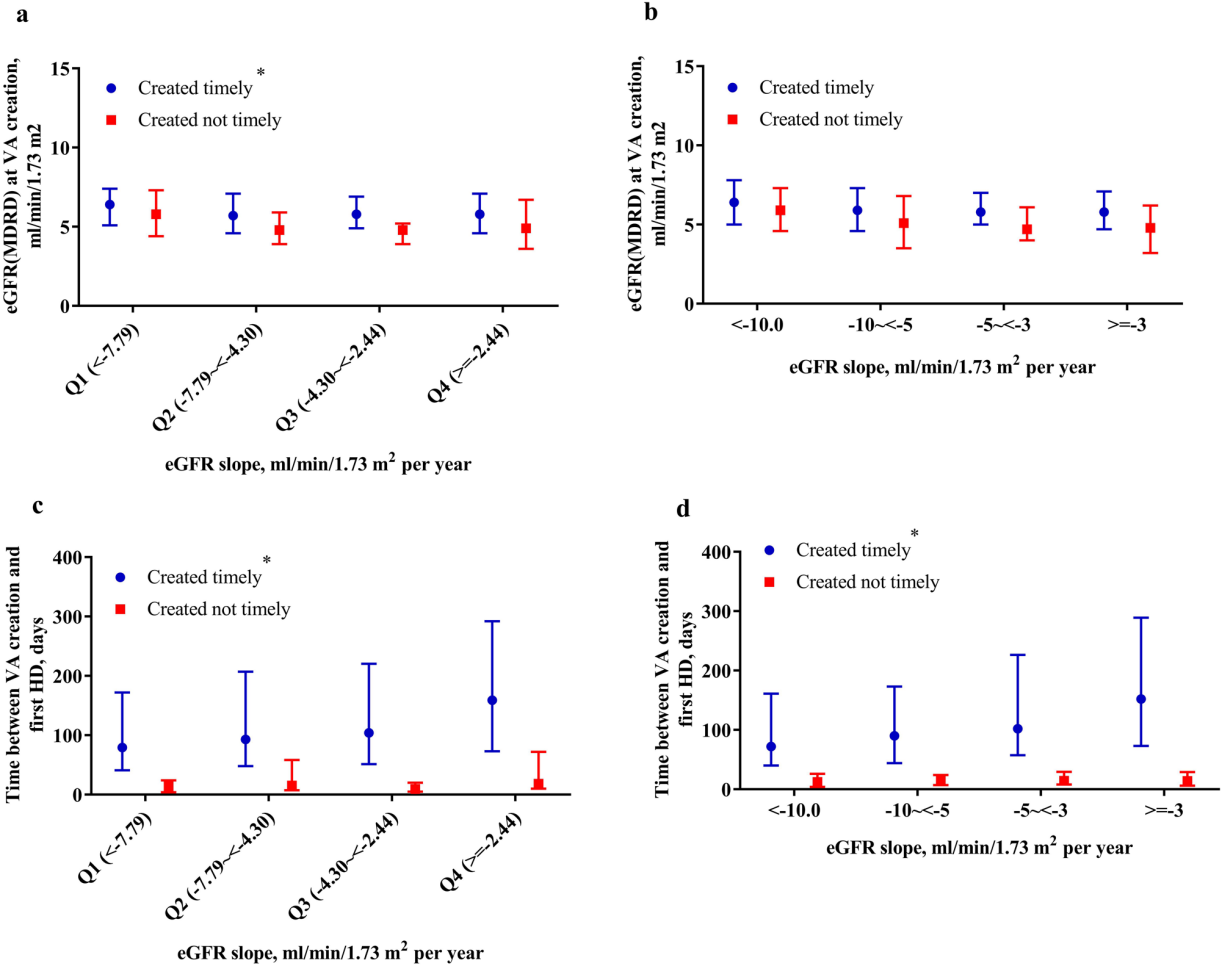
Distribution of estimated glomerular filtration rate (eGFR) and time between vascular access (VA) creation and first hemodialysis in VA created timely and not timely by eGFR slope group. eGFR slope by 75th, 50th and 25th percentiles (**a**,**c**) and eGFR slope by clinical recommendation (**b**,**d**). Asterisk in (**a**) represents the significant difference between slope groups in the group comprising VA created timely (*P* = 0.02), and there was a non-significant difference between slope groups in the group comprising VA created not timely by the Kruskal–Wallis test. Asterisk in (**c**) and (**d**) display the significant difference between slope groups in the group comprising VA created timely (*P* < 0.001), while there was a non-significant difference between slope groups in the group comprising VA created not timely by the Kruskal–Wallis test.

VA was created at a median eGFR of 5.8 min/ml/1.73 m^2^, with a median duration of 3.1 months before HD. Figure 3 reveals the association between eGFR slope and renal function at the time of VA creation. In both the groups comprising VA created timely and not timely, there was a negative association between eGFR slope and eGFR, which was determined either by MDRD or CG formulae at the time of VA creation (Fig. 3b,c, respectively; both *P* < 0.001). However, this negative association disappeared when creatinine (Cr) was used to replace the eGFR (Fig. 3a, P = 0.425). Table 3 reveals the distribution of VA maturation among various eGFR slope ranges. As expected, the fastest-progressing group, whether cut by the first quartile or − 10 ml/min/1.73 m^2^/year, had the lowest percentage of VA created timely, while the remaining groups shared similar success rates. In contrast with the longer duration between VA creation and the first HD as the eGFR slope becomes more flattened in the group comprising VA created timely, eGFRs upon VA creation were not significantly different among various eGFR slope ranges in both the groups consisting of VA created timely and not timely (Fig. 4). The eGFR differences upon VA creation between VA created timely and not timely, at approximately 0.9 ml/min/1.73 m^2^, were not affected by the various eGFR slope groups (Fig. 4a,b), while the time difference between VA creation and the first HD increased as eGFR slope became slower (Fig. 4c,d). Supplementary Figure S1 further displays the distribution of percentages of VA created timely by eGFR slope, with an interval of 2 ml/min/1.73 m^2^/year. We identified that there was a trend whereby slower eGFR slopes were associated with higher VA created timely, even though the middle progression with eGFR slope between − 4 and − 6 ml/min/1.73 m^2^/year had a slightly higher percentage of VA created timely. Table 4 shows the results of the multivariate analysis regarding factors and their interactions associated with VA created timely. After adjusting with covariates, only renal function upon VA creation and eGFR slope were associated with the timely creation of VA. When renal function was expressed by Cr, sex and age upon VA creation were significant predictors for VA created timely. But when the eGFR was used to express renal function upon VA creation, sex and age were no longer significant predictors. Using the third quartile of eGFR slope (− 4.30 ~  < − 2.79 ml/min/1.73 m^2^/year) as a reference, patients with slightly steeper eGFR slope (− 7.79 < − 4.30 ml/min/1.73 m^2^/year) were 30% more likely, albeit not statistically significantly, to have VA created timely. For those with the steepest eGFR slope (< − 10 ml/min/1.73 m^2^/year), the likelihood of having VA created timely was around only 50%. The patients with the flattest eGFR slope (≥ − 2.79 ml/min/1.73 m^2^/year) had similar results to those of the reference group. If we used the cutting ranges recommended by guidelines, consistent findings were produced, as shown in Supplementary Fig. S2.

**Table 3:** Distribution of vascular access created timely, created not timely and not created by eGFR slope.

	VA created timely (n = 676)	VA created not timely (n = 87)	VA not created (n = 224)	*P* value
**eGFR slope by quartile, ml/min/1.73 m**^**2**^** per year, n (%)**				< 0.001
Q1 (< − 7.79)	158 (57.2)	33 (12.0)	85 (30.8)	
Q2 (− 7.79 ~ < − 4.30)	175 (72.9)	16 (6.7)	49 (20.4)	
Q3 (− 4.30 ~ < − 2.44)	172 (71.1)	18 (7.4)	52 (21.5)	
Q4 (≥ − 2.44)	171 (74.7)	20 (8.7)	38 (16.6)	
**eGFR slope by clinical recommendation, ml/min/1.73 m**^**2**^** per year, n (%)**				< 0.001
Group 1 (< − 10)	107 (56.0)	22 (11.5)	62 (32.5)	
Group 2 (− 10 ~ < − 5)	171 (67.1)	22 (8.6)	62 (24.3)	
Group 3 (− 5 ~ < − 3)	164 (71.9)	16 (7.0)	48 (21.1)	
Group 4 (≥ -3)	234 (74.8)	27 (8.6)	52 (16.6)	

The eGFR was calculated using the MDRD equation and the eGFR equation: 186 × serum creatinine^−1.154^ ×age^−0.203^ × 0.742 (if female)^21,22^, and slope was estimated using all eGFRs between the timing upon CKD program enrollment and VA creation or starting hemodialysis—whichever came first. If there were fewer than three eGFR measurements within the aforementioned duration, we would include all eGFR measurements within 1 year prior to VA creation or starting HD—whichever came first.

Significantly different distributions of consequence of vascular access preparation between different eGFR slope groups were tested using the χ^2^ test. A two-tailed value of *P* < 0.05 was considered to be statistically significant.

**Table 4:** Factors predicting vascular access created timely with renal function expressed by Cr, MDRD-eGFR and CG-eGFR.

Variable	Adjusted OR (95% CI)	*P* value	Adjusted OR (95% CI)	*P* value	Adjusted OR (95% CI)	*P* value
**Sex**						
Female	1.00 (reference)					
Male	2.02 (1.23, 3.31)	0.005				
**Age at VA creation**	0.97 (0.95, 0.99)	0.008				
**Renal function at VA creation**						
By Cr (mg/dL)	0.81 (0.74, 0.89)	< 0.001				
By eGFR (MDRD) (ml/min/1.73 m^2^)			1.27 (1.11, 1.46)	0.001		
By eGFR (CG) (ml/min/1.73 m^2^)					1.24 (1.11, 1.39)	< 0.001
**eGFR slope (ml/min/1.73 m**^**2**^ per year)*		0.001^#^		0.008^#^		0.001^#^
Q1 (< − 7.79)	0.37 (0.19, 0.73)	0.004	0.46 (0.24, 0.86)	0.015	0.38 (0.20, 0.72)	0.003
Q2 (− 7.79 ~ < − 4.30)	1.28 (0.61, 2.72)	0.5	1.30 (0.62, 2.72)	0.5	1.22 (0.58, 2.54)	0.6
Q3 (− 4.30 ~ < − 2.44)	1.00 (reference)	–	1.00 (reference)	–	1.00 (reference)	–
Q4 (≥ − 2.44)	0.97 (0.49, 1.93)	0.9	0.95 (0.48, 1.87)	0.9	1.02 (0.52, 2.03)	0.9

MDRD eGFR equation: 186 × serum creatinine^−1.154^ × age^−0.203^ × 0.742 (if female)^21,22^. Cockcroft–Gault eGFR equation: [(140 − age)] × weight × 0.85 (if female)/(72 × serum creatinine), adjusted for BSA/1.73 m^2^. $BSA = weight~_{{{\text{kg}}}}^{{0.425}} \times height~_{{{\text{cm}}}}^{{0.725}} \times 0.007184$^22,23^.

Multiple logistic regression included the variables of sex, age at shunt creation, marital status, year of education, occupation, smoking, diabetes mellitus, renal function at chronic kidney disease (CKD) program enrollment, renal function at shunt creation, eGFR slope, CKD care program duration, and time period when VA was created. Here are only shown the significant variables by backward selection in this multivariable logistic regression model. A two-tailed value of *P* < 0.05 was considered to be statistically significant.

*VA* vascular access, *OR* odds ratio, *CI* confidence interval, *Cr* creatinine, *eGFR* estimated glomerular filtration rate, *MDRD* modification of diet in renal disease, *CG* Cockcroft–Gault, *BSA* body surface area.

*Quartile of eGFR slope, using eligible eGFRs calculated by MDRD equation.

^#^*P* value for linear trend.

## Discussion

This is the first report showing, through the multidisciplinary CKD program, that a planned initiation of maintenance HD without a temporary catheter can be constantly achieved in more than 70% of all enrolled patients. The median days between VA creation and the first dialysis constituted almost 3 months for those with timely creation, while the mean days constituted around 6 months. In addition to a higher eGFR at the time of VA creation, eGFR slope played an important role in our findings with regard to VA created timely. Fast progression of CKD led to much less VA created timely, but slow-progression groups did not achieve as high a percentage of VA created timely as that achieved by the middle-progression group. Our findings in this observational study could provide a practical recommendation when determining the time of VA creation in the care of late CKD patients.

Registry data indicating timely VA creation varies among countries from less than 20% to almost 80%^7,24–26^. Given the many advantages of not using temporary central catheters to initiate dialysis but the unsatisfactory results worldwide^27,28^, a strategy with which to increase VA created timely should be well examined in every CKD care unit. To create VA timely for the initiation of dialysis, referral in time, regular follow-up of CKD progression, conducting shared decision making on the choice of RRT modality, and determining the time of VA creation are all needed, in that order^14,18,29^. Thus, it seems very reasonable to consider timely VA creation to be a potential candidate for the quality index of pre-dialysis CKD care. Through the multidisciplinary CKD program, our cohort showed a continuous improvement in the proportion of patients starting HD with distal VA of more than 70% and whose results had been consistent for the 6 years running up to 2016. A few HD centers have a similar performance at such a percentage or even higher^7,30^, which suggests that the figure of 70% might be an achievable goal for many other centers to reach.

The European Best Practice Guidelines recommend arteriovenous fistula creation at least 2–3 months prior to HD, whereas the NKF-KDOQI used to suggest at least 6 months prior to HD, but recently have recommended that physicians begin VA creation preparation with patients when entering late CKD stage 4, as it is impractical to arrange VA creation based on predicting the time when HD is needed^11,31,32^. Taiwan has relatively late dialysis initiation, with an eGFR of 5.9 ml/min/1.73 m^2^ in 2017^33^. Amongst this 15-year cohort, patients received VA creation at an eGFR of 4.1–7.1 (Q1–Q3) ml/min/1.73 m^2^ and started HD at an eGFR of 3.4–5.5 (Q1–Q3) ml/min/1.73 m^2^, with the median duration between VA creation and dialysis starting at approximately 3 months. The negative association between eGFR slope and the eGFR upon VA creation demonstrated that nephrologists in our program had used eGFR slope as a guide in determining when VA creation was needed. The high successful rate of VA creation timely implied that it is practical to prepare VA creation based on predicting the onset of dialysis. The present NKF-KDOQI guideline recommendation of starting VA creation when entering CKD stage 4 with an eGFR of 15–20 ml/min/1.73 m^2^ raises concern surrounding the duration between VA creation and initiation of dialysis would become much longer. The longer duration referred to above may increase risks such as having VA fail and death before use^29^, not to mention having a higher cardiac burden from VA shunting. In addition, unit would consume much more human resource in convincing patients for earlier schedules. In brief, we used our clinical results to support the contention that using eGFR slope for preparing VA creation by predicting the occurrence of HD is feasible with a high probability of success. The guidelines could give more precise individual suggestions regarding the timing of VA creation with putting eGFR slope into consideration.

Fistula first is our policy for a very long time, with the distribution of AVF (85%) and AVG (15%) in our study cohort of 2003–2016 (Fig. 2). In our hospital, the surgeon is the one who suggests whether fistula or graft should be created based on the vessel condition, and the nephrologist only suggests permanent catheter insertion if a short life span or temporary dialysis duration is expected. Again, our data support this practice pattern as the AVG had the same distribution in both created timely and not timely groups. In our practice, we did not arrange AVG creation for the reason of early cannulation. A temporary or permanent catheter will be used once early cannulation is required. With the introduction of ESKD Life-Plan by KDOQI in 2019^11^, it recommends taking VA creation and care as a continuous process from predialysis until the whole ESKD. For the first access creation, a patient-centered approach for dialysis modality choice, vessel condition evaluation, VA creation, and maturation for cannulation have all been well documented. Fistula-First might be no longer suited to everyone who chose HD. When preparing the distal VA in the elderly, the more inferior vessel quality, higher comorbidity burden, and shorter life expectancy even further addressed the necessity of ESKD Life-Plan in this population. To complete all the processes as mentioned above, starting the preparations at CKD stage 4 is encouraged. We believe the eGFR slope can help the care team take the comprehensive steps in VA creation suggested by ESKD Life-Plan at proper timings regarding local practice patterns.

The eGFR rather than creatinine, like detecting CKD^34^, should be used to make the decision regarding VA creation simpler according to our model suggestion. In addition to being a surrogate of renal outcomes^35^, the role of eGFR slope in timely VA preparation should be addressed how it could predict the initiation of dialysis. When exhibiting an eGFR slope steeper than − 10 ml/min/1.73 m^2^/year, patients would have VA creation scheduled much earlier based on prior recommendations^11^; now we offer the evidence which can be used to support this expert consensus. In contrast to our retrospective observation, Al-Balas et al. have published a study on the optimal timing of predialysis AVF creation with a prospective approach^29^. With night-tenth of AVF creation at eGFR higher than 10 ml/min/1.73 m^2^, only 47% of the participants used this permanent access on the first HD session, and 15% died before dialysis initiation. They can't find any factors predicting the use of mature AVF on dialysis initiation, but the eGFR at AVF creation, diabetes mellitus, eGFR slope, and degree of proteinuria are associated with HD initiation within two years after the AVF creation. In addition, the non-significant difference of the eGFR upon VA creation among the remaining various eGFR slope groups implied, even with satisfactory success probability, that other factors may contribute to the prognosis. We speculate that patients’ decision making might play a role in delayed VA creation for the following findings: firstly, the very short duration before the first HD in VA created not timely among every eGFR slope group, secondly, that the eGFR differences upon VA creation between VA created timely and not timely were fixed at various eGFR slopes, and, thirdly, that VA created not timely had a lower eGFR than did VA created timely when starting the first HD. Then, starting VA preparation earlier may have few effects on improving the probability of success.

There were several limitations in our study, especially due to its retrospective and observational study design. Firstly, the generalization of our findings may be limited regarding our VA created at a relatively low eGFR level and fast surgical assistance, and we only included those late CKD patients under multidisciplinary CKD program for more than 6 months. However, the consistent high timely creation of VA in our study, and in certain other studies, might support the notion that the majority of CKD teams can initiate planned HD through distal VA with a certain probability of success. Secondly, how the patients made their decision was not measured, which could be a main factor contributing to the timely creation of VA. There was no difference in both eGFR slope before HD and eGFR values upon starting HD between patients receiving VA creation and those not; this implied that patients’ refusal of VA creation might play a determining role for those with VA not created. Thus, including only those having VA creation within analysis means that the results are less influenced by patients’ compliance. Thirdly, the disparity of clinical experience among nephrologists is another unmeasurable factor when running the prediction model. Finally, factors other than eGFR slope, such as the degree of proteinuria, are not included in the analysis to explain the timely VA creation. Although the eGFR slope itself is a very dominant predictor of CKD progression, several studies consider it as CKD progression; other potential indexes are still having the chance for better prediction power. Nevertheless, the standard procedures within the multidisciplinary care did mean that the majority of in-program patients received similar management. Although the eGFR slope itself is a very dominant predictor of CKD progression, several studies consider it a CKD progression^35,36^, more potential indexes are needed for better prediction power.

## Conclusion

Our study revealed that, through the multidisciplinary CKD program, participants can constantly have timely VA creation of more than 70%. Factors associated with timely VA creation include receiving VA creation at a higher eGFR and flattening the eGFR slope. The eGFR slope should be highlighted when advanced CKD patients are scheduled for VA creation.

## Supplementary Information

Supplementary Figure S1.

Supplementary Figure S2.

Supplementary Figure S3.

Supplementary Information.

## Abbreviations

AVF: Arteriovenous fistula
AVG: Arteriovenous graft
CG: Cockcroft and gault
CKD: Chronic kidney disease
eGFR: Estimated glomerular filtration rate
HD: Hemodialysis
MDRD: Modification of diet in renal disease
NHI: National Health Insurance
NKF-KDOQI: National Kidney Foundation-Kidney Disease Outcomes Quality Initiative
RRT: Renal replacement therapy
VA: Vascular access
