# Supplementary figures and images for: Association of glomerular filtration rate slope with timely creation of vascular access in incident hemodialysis
Source: Sci Rep. 2021 Jun 23;11:13137. doi: 10.1038/s41598-021-92359-w (PMC8222220; doi:10.1038/s41598-021-92359-w)

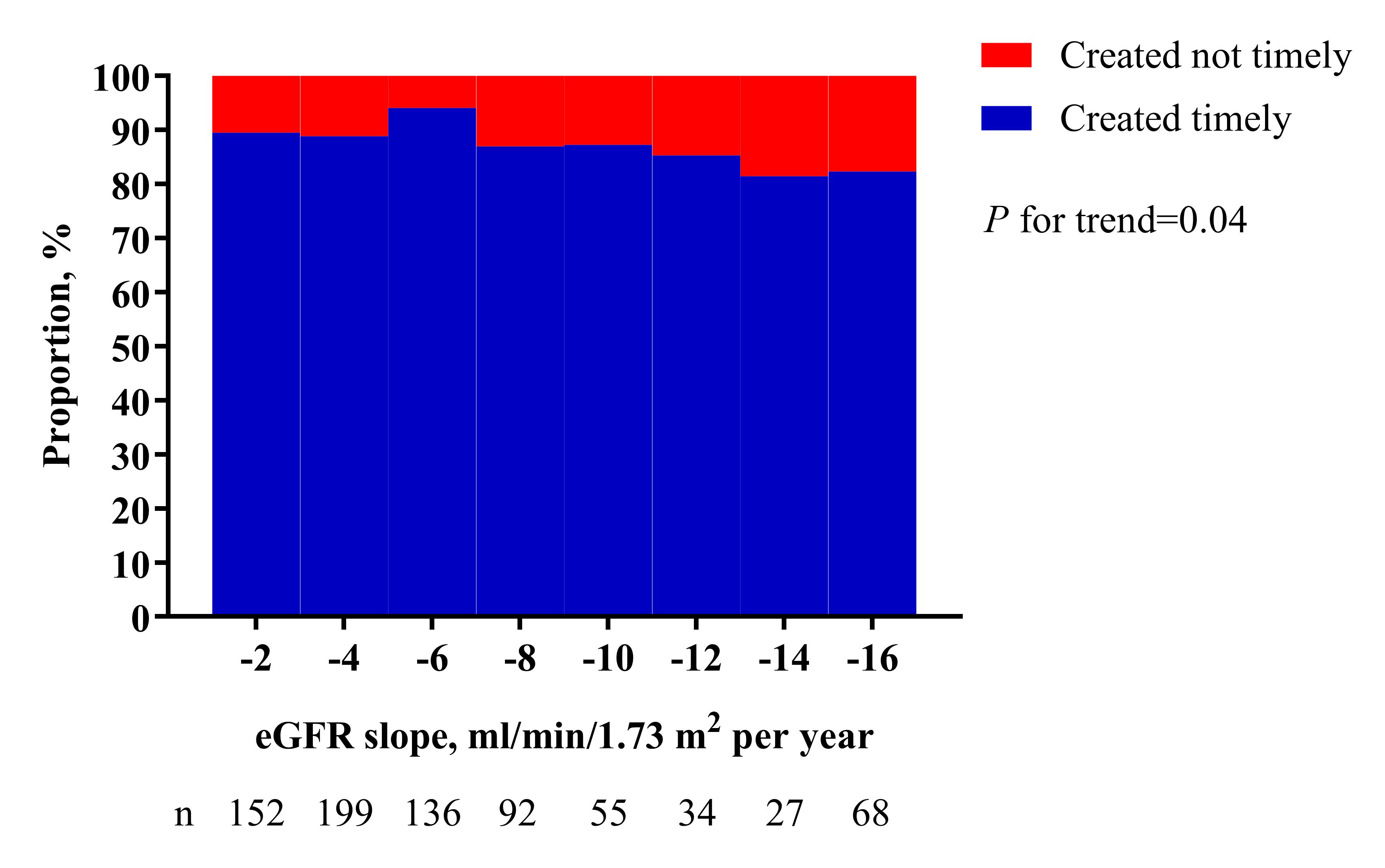

Supplement: Supplementary file 1 — Supplementary Figure S1. [file 41598_2021_92359_MOESM1_ESM.jpg]

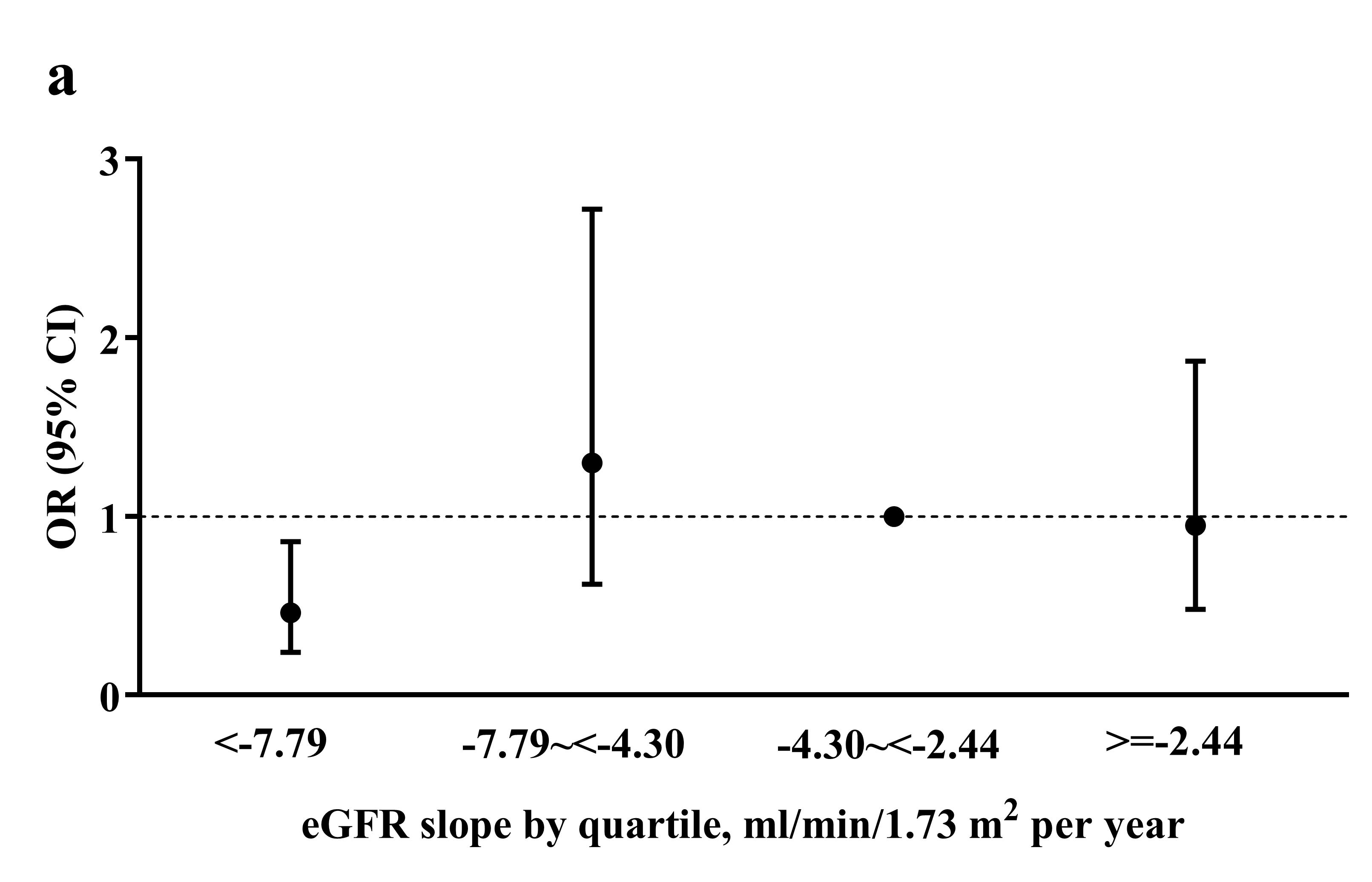

Supplement: Supplementary file 2 — Supplementary Figure S2. [file 41598_2021_92359_MOESM2_ESM.jpg]

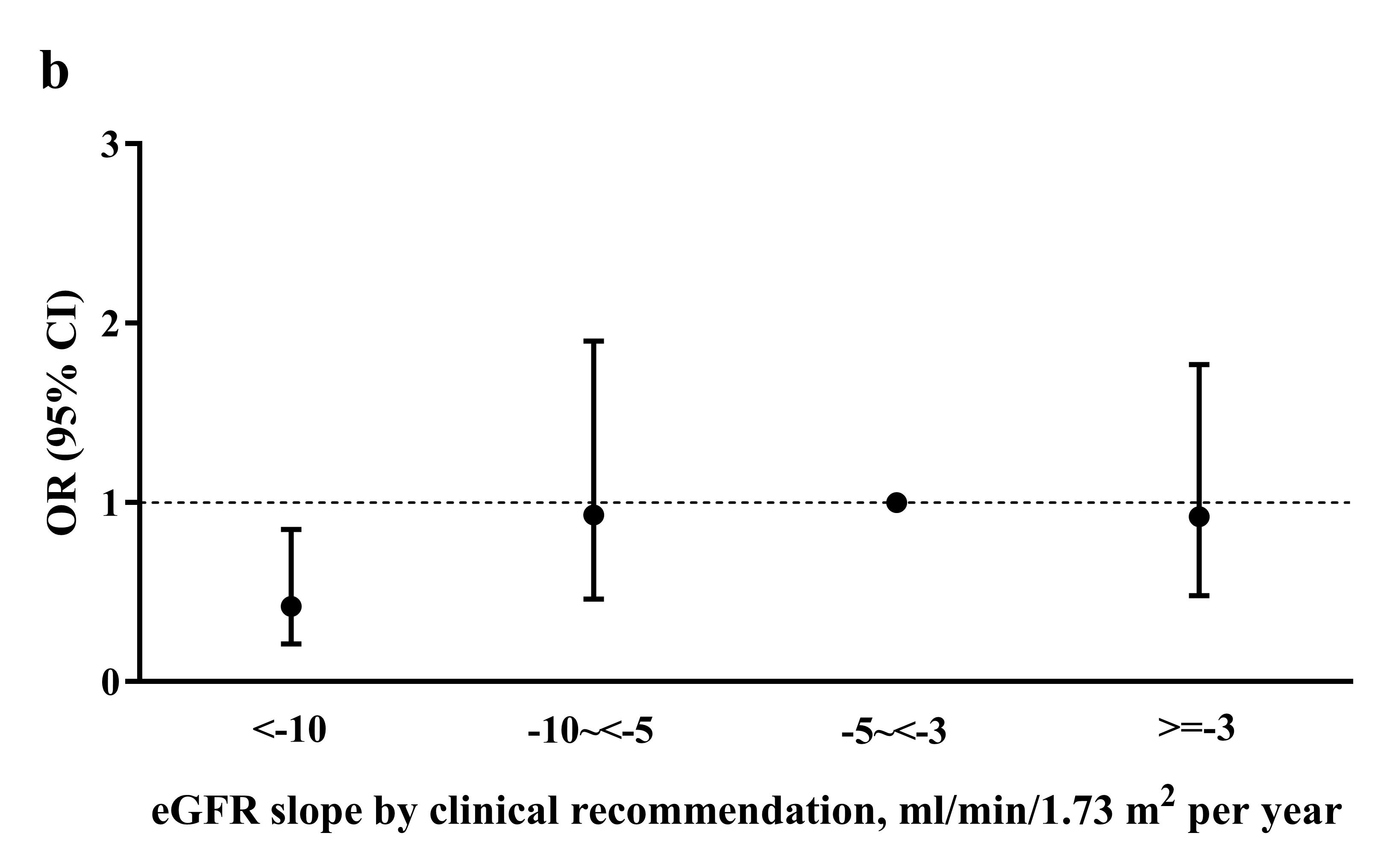

Supplement: Supplementary file 3 — Supplementary Figure S3. [file 41598_2021_92359_MOESM3_ESM.jpg]
