# Supplementary material for: Association of glomerular filtration rate slope with timely creation of vascular access in incident hemodialysis
Source: Sci Rep. 2021 Jun 23;11:13137. doi: 10.1038/s41598-021-92359-w (PMC8222220; doi:10.1038/s41598-021-92359-w)
Supplement: Supplementary file 4 — Supplementary Information. [file 41598_2021_92359_MOESM4_ESM.docx]

**eGFR Slope and Timely Creation of Vascular Access: Is Earlier Creation Necessary for All Late CKD?**

Lee-Moay Lim, MD.^1,2 #^, Ming-Yen Lin, Ph.D.^1,3 #^, Shang-Jyh Hwang, M.D.^1,3^, Hung-Chun Chen, M.D., Ph.D.^1,3^, and Yi-Wen Chiu, M.D.^1,3^*

^1^Division of Nephrology, Department of Internal Medicine, Kaohsiung Medical University Hospital, Kaohsiung, Taiwan

^2^Faculty of Medicine, College of Medicine, Kaohsiung Medical University, Kaohsiung, Taiwan

^3^Faculty of Renal Care, College of Medicine, Kaohsiung Medical University, Kaohsiung, Taiwan

^#^The authors are equal contributors

*Corresponding authors

Yi-Wen Chiu, MD, email: [chiuyiwen@kmu.edu.tw](mailto:chiuyiwen@kmu.edu.tw) / [chiuyiwen@gmail.com](mailto:chiuyiwen@gmail.com)

100 Tzyou First Road,

Sanmin District, Kaohsiung City 80708, Taiwan

Tel.: 886-7-3121101-7351-11; Fax: 886-7-3228721

**Short running title:** eGFR slope and vascular access creation.

Abbreviations: AVF, arteriovenous fistula; AVG, arteriovenous graft; CG, Cockcroft and Gault; CKD, chronic kidney disease; eGFR, estimated glomerular filtration rate; HD, hemodialysis, MDRD, Modification of Diet in Renal Disease; NHI, National Health Insurance; NKF-KDOQI, National Kidney Foundation-Kidney Disease Outcomes Quality Initiative; RRT, renal replacement therapy; VA, vascular access.

Table S1. STROBE Checklist for cross-sectional studies.

|  | Item No | Recommendation | Check |
| --- | --- | --- | --- |
| **Title and abstract** | 1 | (*a*) Indicate the study’s design with a commonly used term in the title or the abstract | The sentence, “This retrospective study enrolled chronic kidney disease (CKD) patients, ~” on the 2^nd^ raw in the abstract. |
|  |  | (*b*) Provide in the abstract an informative and balanced summary of what was done and what was found | Lines 6-12 in the abstract. |
| Introduction | | |  |
| Background/rationale | 2 | Explain the scientific background and rationale for the investigation being reported | We have described it in lines 1 to 56 on page 4. |
| Objectives | 3 | State specific objectives, including any prespecified hypotheses | We have described it in lines 15 to 22 on page 5. |
| Methods | | |  |
| Study design | 4 | Present key elements of study design early in the paper | It has been described in line 8 on page 15. |
| Setting | 5 | Describe the setting, locations, and relevant dates, including periods of recruitment, exposure, follow-up, and data collection | We have described these in the first two paragraphs on page 15. |
| Participants | 6 | (*a*) Give the eligibility criteria, and the sources and methods of selection of participants | They have been described in the first paragraph of the results section on page 6. |
| Variables | 7 | Clearly define all outcomes, exposures, predictors, potential confounders, and effect modifiers. Give diagnostic criteria, if applicable | They have been described in the 3^rd^-5^th^ paragraph of the methods section on pages 15-17. |
| Data sources/ measurement | 8* | For each variable of interest, give sources of data and details of methods of assessment (measurement). Describe comparability of assessment methods if there is more than one group | We have described in the 3^rd^-5^th^ paragraph of the methods section on pages 15-17. |
| Bias | 9 | Describe any efforts to address potential sources of bias | We have addressed on limitation in line 35, page 13 to line 20, page 14. |
| Study size | 10 | Explain how the study size was arrived at | They have been described in the first paragraph of the results section on page 6. |
| Quantitative variables | 11 | Explain how quantitative variables were handled in the analyses. If applicable, describe which groupings were chosen and why | N/A |
| Statistical methods | 12 | (*a*) Describe all statistical methods, including those used to control for confounding | Please refer to the section on statistical analysis on page 17. |
|  |  | (*b*) Describe any methods used to examine subgroups and interactions | The interaction test is regularly performed when modeling. There was no any interaction term that should be included in the final model. |
|  |  | (*c*) Explain how missing data were addressed | We have explained it in figure 1. |
|  |  | (*d*) If applicable, describe analytical methods taking account of sampling strategy | NA |
|  |  | (*e*) Describe any sensitivity analyses | NA |
| Results | | |  |
| Participants | 13* | (a) Report numbers of individuals at each stage of study—eg numbers potentially eligible, examined for eligibility, confirmed eligible, included in the study, completing follow-up, and analysed | NA |
|  |  | (b) Give reasons for non-participation at each stage | NA |
|  |  | (c) Consider use of a flow diagram | We have exhibited it in figure 1. |
| Descriptive data | 14* | (a) Give characteristics of study participants (eg demographic, clinical, social) and information on exposures and potential confounders | We have displayed these in table 1. |
|  |  | (b) Indicate number of participants with missing data for each variable of interest | We have labeled this information on the footnote of tables. |
| Outcome data | 15* | Report numbers of outcome events or summary measures | NA |
| Main results | 16 | (*a*) Give unadjusted estimates and, if applicable, confounder-adjusted estimates and their precision (eg, 95% confidence interval). Make clear which confounders were adjusted for and why they were included | We have represented these in Tables 3 and 4. |
|  |  | (*b*) Report category boundaries when continuous variables were categorized | We have reported in Tables 3 and 4. |
|  |  | (*c*) If relevant, consider translating estimates of relative risk into absolute risk for a meaningful time period | NA |
| Other analyses | 17 | Report other analyses done—eg analyses of subgroups and interactions, and sensitivity analyses | We did not do analyses of subgroups due to insignificance by interation analysis. |
| Discussion | | |  |
| Key results | 18 | Summarise key results with reference to study objectives | Please refer to the first paragraph of the discussion on page 10. |
| Limitations | 19 | Discuss limitations of the study, taking into account sources of potential bias or imprecision. Discuss both direction and magnitude of any potential bias | We have addressed on limitation in line 35, page 13 to line 20, page 14. |
| Interpretation | 20 | Give a cautious overall interpretation of results considering objectives, limitations, multiplicity of analyses, results from similar studies, and other relevant evidence | Please refer to 2-4 paragraphs on pages 10-13. |
| Generalisability | 21 | Discuss the generalisability (external validity) of the study results | Please refer to the first point of limitation on page 13. |
| Other information | | |  |
| Funding | 22 | Give the source of funding and the role of the funders for the present study and, if applicable, for the original study on which the present article is based | We have appropriately declared in financial disclosure. |

*Give information separately for exposed and unexposed groups.

**Note:** An Explanation and Elaboration article discusses each checklist item and gives methodological background and published examples of transparent reporting. The STROBE checklist is best used in conjunction with this article (freely available on the Web sites of PLoS Medicine at http://www.plosmedicine.org/, Annals of Internal Medicine at http://www.annals.org/, and Epidemiology at http://www.epidem.com/). Information on the STROBE Initiative is available at www.strobe-statement.org.

**Figure legend**

Fig. S1. Proportion of timely created vascular access by estimated glomerular filtration rate (eGFR) slope in the interval of 2 ml/min/1.73 m^2^/year. Significant trends in the proportions across different hemodialysis onset years (*P*=0.04) are found by using the Cochran–Armitage trend test.

Fig. S2. Adjusted odds ratio (OR) of vascular access created timely by estimated glomerular filtration rate (eGFR) slope grouped by quartile (2a) and clinical recommendation (2b).
